# *“It was really poor prior to the pandemic. It got really bad after”*: A qualitative study of the impact of COVID-19 on prison healthcare in England

**DOI:** 10.1186/s40352-023-00212-1

**Published:** 2023-02-07

**Authors:** Lucy Wainwright, Sarah Senker, Krysia Canvin, Laura Sheard

**Affiliations:** 1 Department of Health Sciences, University of York, Seebohm Rowntree Building, Heslington, York, YO10 5DD UK; 2 Leeds Institute of Health Sciences, School of Medicine, University of Leeds, Worsley Building, Leeds, LS2 9JT UK

**Keywords:** Healthcare, Pandemic, Prisoners, Healthcare staff, Health inequalities, Conflict, Framework analysis

## Abstract

**Background:**

The impact of COVID-19 has been exceptional, particularly on the National Health Service which has juggled COVID affected patients alongside related staff shortages and the existing (and growing) health needs of the population. In prisons too, healthcare teams have been balancing patient needs against staffing shortfalls, but with additional strains unique to the prison population. Such strains include drastic lockdown regimes and prolonged isolation, the need to consider health alongside security, known health inequalities within prisoner groups, and an ageing and ethnically diverse population (both groups disproportionately affected by COVID). The aim of this paper is to contribute to emerging research on the impact of COVID-19 on prison healthcare.

**Methods:**

We conducted 44 in depth interviews (over phone or video) across three groups: prison leavers, healthcare staff and decision makers, between July and December 2021. Framework analysis was undertaken.

**Results:**

Three themes were found. First, we found that Covid-19 had a significant impact on prison healthcare which involved reduced access and changes to how healthcare was delivered. This affected the health of prisoners by exacerbating existing conditions, new conditions being undiagnosed and mental health needs increasing. Second, the pandemic impacted on healthcare staff through creation of stress, frustration and exhaustion due to minimal staffing levels in an already under-resourced system. Third, an emerging conflict was witnessed. People in prison felt neglected regarding their healthcare needs but staff reported doing the best they could in an unprecedented situation. Healthcare staff and decision makers felt that prison healthcare was seen as a poor relation when compared with healthcare in the community, with no extra resource or staffing for Covid-19 testing or vaccinations.

**Conclusion:**

The Covid-19 pandemic has significantly impacted almost all aspects of prison healthcare in the UK. This includes delivery of healthcare by staff, receipt of it by people in prison and the management, planning and commissioning of it by decision makers. These three groups of people were all affected detrimentally but in vastly different ways, with some participants describing a sense of trauma. Health needs that were exacerbated or went unmet during Covid urgently need to be addressed in order to reduce health inequalities. In order for welfare and wellbeing to be maintained, and in some cases repaired, both prisoners and staff need to feel heard and recognised.

## Background

In the years preceding the pandemic, the United Kingdom’s National Health Service (NHS) was already under scrutiny following years of inadequate funding alongside staff shortages and rising public need. In 2020, the NHS was around 1 year into its 10-year ‘long term plan’ for a more sustainable future, given the growing and changing needs of the population (NHS, 2019). However, the emergence of the pandemic in early 2020 changed the landscape dramatically, and likely for many years to come (Thorlby et al., 2021). In the community, the pandemic meant many non-urgent appointments were postponed or cancelled to reduce social interaction, and telephone and video appointments were utilised widely (Shaw et al., 2021). There was significant redeployment of staff to care for COVID-19 patients or provide alternative services, as well as self-isolation, infection and also death within the staff group, leaving a shortage in multiple clinics (Sykes & Pandit, 2021; Vindrola-Padros et al., 2020). The result has been unavoidable delays to routine care and a rapidly growing backlog of unmet need with hospital waiting lists exceeding 5.6 million in July 2021 (Gardner & Fraser, 2021). However, there has also been concern raised for the psychological wellbeing of staff across the NHS as a result of the pandemic, with references being made to the fallout of previous health crises such as the 2002–2004 SARS epidemic (Cabarkapa et al., 2020). The NHS in the community faced a collision between an already stretched service and the novel demands of the pandemic.

Ostensibly, prison healthcare in England and Wales faced a similar collision, with well-documented staffing and resource tensions nestled amongst insufficient funding (National Audit Office, 2020). On the announcement of the first national lockdown, prisons went into ‘command mode’^1^ and operated ‘Exceptional Delivery Models’ (Brennan, 2020) to radically restrict prisoner movement to contain the virus. Similar to the community, prison healthcare changed overnight with reduced face-to-face contact, increased isolation and the introduction of alternative models of delivery (Canvin & Sheard, 2021).

The impact of this collision was intensified, however, by the unique nature of the prison setting and population within which healthcare operates; its ‘legacy factors’ (Canvin & Sheard, 2021). The close proximity of staff and residents in ‘total institutions’ (Goffman, 1968) such as prisons makes the spread of infection a significant risk. Features common in the prison estate in England, including the use of buildings constructed in the late nineteenth century, overcrowding, multiple occupancy of cells, and under-resourcing present a challenge to social distancing, handwashing and decontamination. Infection control is also constrained by the prison regime and shared use of facilities and physical space. Inadequate funding and concerns over bringing alcohol-based products into the prison affected the availability of sanitiser in prisons (Suhomlinova et al., 2021) despite clear transmission risks posed by the shared use of wing-based telephones (Prison Reform Trust, 2021). The need to consider security simultaneously with health undoubtedly limits what healthcare departments are able to do and intensifies the potential impact of COVID-19 on prisoners.

Other legacy factors include the state of prisoners’ health prior to the pandemic and their vulnerability to COVID-19. For example, physical health is poorer in prisoners as compared with the general population and prisoners face distinct health inequalities. The mortality rate for prisoners is 50% higher than the rest of the population, and many prisoners have been found to have the biological characteristics of those who are 10 years older (House of Commons, 2018). Across the board, COVID-19 outcomes have also been poorer for those who are from black and minority ethnic communities, as well as for those who are older (Public Health England, 2020). This is particularly relevant to this work since prisons are both ethnically diverse and house an ageing population (Prison Reform Trust, 2022). Seventy-one percent of women and 47% of men in prison experience mental health problems (Prison Reform Trust, 2022), and self-harm incidents were at a record high in 2019, up 14% from the previous 12 months (House of Commons, 2020).

In April 2020, modelling conducted by HM Prison and Probation Service (HMPPS) and Public Health England (PHE) suggested that 2700 prisoners might die from COVID-19 if no action was taken to reduce contact in prisons (O’Moore, 2020). Fortunately, numbers did not reach those levels, yet those in prison have still been disproportionately affected by COVID-19 despite stringent measures. Data up to the end of December 2020 showed 75 COVID-19 cases per 1000 population in prison, compared with 46 per 1000 in England and Wales overall. Data up to early 2021 showed that prisoners experienced three times the death rate from COVID-19 compared with people of the same age and sex in the general population (Edge et al., 2021).

Within prisons, the impact of the pandemic was not only felt by those who contracted the virus but by all prisoners incarcerated at that time. Exceptional delivery models meant significant and prolonged time in cell, for up to 23.5 hours a day (or 24 hours a day for those self-isolating). For those in a single cell, this led to a palpable isolation, and for those whose isolation was not absolute (i.e. in a shared cell), negative effects were still significant (Suhomlinova et al., 2021). Exceptional delivery models also imposed restrictions on external visitors including family, friends, external service providers and support charities. There was a distinct lack of movement, both within and between prisons, as well as a lack of access to structured activities such as offender rehabilitation courses, employment and education. Crucially too, the ‘opening up’ of prisons did not happen at the same pace as it did in the community and, as of February 2022, many prisons are still operating under restricted regimes with recurrent breakouts of COVID-19 (Thorlby et al., 2021).

Reporting in the grey literature suggests that both the physical and mental health of people in prison has deteriorated during the pandemic. Declines to mental health are likely to be related to prolonged in cell confinement (Wainwright & Gipson, 2020) and declines to physical health related to a corresponding lack of physical activity as well as challenges in accessing appointments (Gipson & Wainwright, 2020). Recent work, involving peer researchers, which engaged 1400 prisoners across 11 prisons indicated that 85% of surveyed prisoners were confined to cells for 23 hours for the majority of the lockdown period. 59% of surveyed prisoners had not had a single family visit during the Covid lockdown. Standard wellbeing screening tools suggested depression and anxiety scores were almost 5 times higher than the standard for the general population and more than 1 out of 3 prisoners were scoring at the level of “severe anxiety disorder” indicating high levels of post-traumatic stress (User Voice, 2022).

The present study qualitatively explored the impact that Covid-19 had on the provision, delivery and receipt of prison healthcare in England through in-depth interviews. This afforded consideration to the subsequent implications for patient health from the perspective of those who lived and worked in prisons during this time. In this paper, we describe how and in what ways has prison healthcare changed in response to COVID-19 and how these changes were experienced. We conclude by considering how our findings can influence and inform the commissioning and delivery of prison healthcare in the future.

## Methods

### Study design and setting

We conducted an inductive qualitative study, using semi-structured, in-depth interviews with people who had lived or worked in prisons or occupied a strategic role relating to prisons during the pandemic. The study was predominantly conducted in England and all interviews were undertaken in the community. No interviews were conducted in prisons because we considered it unethical to enter the prison estate during the pandemic to recruit or interview participants face-to-face when data could be collected via remote digital means in the community.

We convened a lived experience steering group composed of prison leavers who are members of the Prison Reform Trust’s Prisoner Policy Network and who had been in prison during the pandemic. The purpose of the group was to act as ‘a critical friend’, advising us on content, direction and interpretation of fieldwork. The group met three times online over a 12 month period.

Ethical approval was received from the National Research Committee (NRC) for the prison and probation service, in April 2021, to conduct this study (reference 2021–034). It received additional approval from the University of York Health Science Research Governance Committee (reference HSRGC/2021/448/F: COVID Prison Healthcare Study) in May 2021.

### Sampling and recruitment

We aimed to recruit three types of participants: (a) prison leavers (who had been in prison during the pandemic); (b) front line healthcare staff (who had worked for the prison healthcare service during the pandemic); and, (c) decision-makers (any professional involved in senior level decision-making regarding prison healthcare during the pandemic, e.g., NHS Commissioners, Public Health England senior management and Prison Governors). We strived to include a diversity of demographics including geographical location, range of prisons that people had lived or worked in, age, gender and ethnicity.

We identified healthcare staff and decision-makers by contacting healthcare providers, prison governors and health and justice commissioners from the research team’s existing networks. Information was cascaded through those contacted, also using global email lists. We invited prison leavers via charities, agencies and services which the research team were regularly engaging with. We distributed information sheets to all eligible participants. This opportunistic sampling then progressed to snowball sampling whereby those who participated shared the opportunity with their colleagues. We attended meetings populated by a range of healthcare providers including strategic and operational staff to promote the study and invite participation.

As the study progressed, we monitored the diversity of our sample to ensure we heard from both males and females, a range of prisons, and a range of healthcare professions. The study was almost entirely focused on England. We interviewed one decision maker who worked in Wales. When inviting prison leavers, we emphasised that declining to participate would not affect future healthcare provision. Prison leavers who took part received a £20 voucher as a token of appreciation, and were advised of this in the information sheet, before deciding to contribute.

### Data collection and analysis

We collected all data between July 2021 and December 2021 via video or telephone calls. Given the remote nature of the interview, before the interview commenced, verbal informed consent was obtained from each participant and audio-recorded. All interviews were digitally-recorded (with permission) using an encrypted device and transcribed verbatim. Interviews ranged from between 30 to 90 minutes.

Two researchers (joint first authors) conducted interviews with participants from all three groups. Both the interviewers have expertise in undertaking qualitative research regarding prison healthcare and criminal justice, and extensive experience of interviewing people who currently live in prison as well as those who have left.

All interviews were conducted using a topic guide to ensure consistency across participants; however, the format was flexible to allow participants to voice what they considered important. The topic guides differed for each of the three participant groups, according to whether they were a prison leaver, healthcare professional or decision-maker. However, all interviews began with an opportunity to discuss healthcare *before* March 2020. The interview schedule then took a narrative approach to walk through healthcare experiences from March 2020 onwards, focusing on the changes that occurred within healthcare, how those decisions were made, communicated or experienced, the perceived impact of these changes, lessons learned and how healthcare has changed moving forwards. We also asked which prisons participants had experience of during the pandemic.

We undertook a framework analysis (Gale et al., 2013). Data analysis involved a process of organising the data, descriptive coding, charting the data and then interpretation. Both researchers read all the transcripts of the interviews conducted by the other researcher to gain insight and knowledge of the whole dataset. Transcripts were coded and several ‘analysis sessions’ were held where the research team (including the third and last authors) came together to discuss analysis, structure the emergent findings and refine content after having read the transcripts.

## Results

We interviewed 44 individuals in total for this study across the three groups. Fifteen prison leavers, 15 front-line healthcare staff and 14 decision-makers (Table 1).

**Table 1:** Participant characteristics

Participant group	Prison leavers	Healthcare staff	Decision-makers
Male	12	8	5
Female	3	7	9
Age			
*20s*	2	0	0
*30s*	4	2	1
*40s*	5	3	4
*50s*	3	7	4
*Not stated*	1	3	5
Ethnicity			
*White British*	9	14	12
*Black African*	2	1	1
*Black Caribbean*	1	0	0
*African Caribbean*	0	0	1
*Asian*	2	0	0
*Indian*	1	0	0
Pre-existing health condition			
*Disability or long-term condition*	8	2	0
*None*	7	13	14
Prison region			
*London & South East*	6	1	3
*South West*	2	0	1
*Midlands*	0	0	3
*East Midlands*	1	0	1
*West Midlands*	1	5	1
*North*	0	2	1
*North West*	2	2	2
*North East*	3	0	1
*East*	0	2	1
*Not known*	0	2	0
Sentence length			
*1 y or less*	1	–	–
*1–2 y*	5	–	–
*3–4 y*	2	–	–
*4–5 y*	2	–	–
*6–9 y*	0	–	–
*10–14*	4	–	–
*Not known*	1	–	–
Length of service			
*< 4*	–	2	3
*4–10*	–	2	0
*> 10*	–	5	1
*Not stated*	–	5	10

The prison leaver group included 12 males and three females aged between 24 and 56 years. We recorded the most recent prison participants had been in during the pandemic and this included 12 different prisons across England.^2^ Eight prison leavers considered themselves to have a disability or long-term health condition and mentioned high blood pressure, diabetes, COPD, sports injury, Aspergers, and mental health problems. They had served prison sentences of varying lengths (ranging from one to 14 years), had started and ended their sentence at various points, and some had been in prison multiple times before the pandemic (which was useful as they had experiences of healthcare pre-COVID-19). Participants also varied in terms of how long they had been released from prison.

Front-line healthcare staff included seven females and eight males aged between 30 and 58. Just one staff participant described himself as Black African, the rest were White British. Two stated they had a disability. The length of service in prison healthcare ranged from 2 to 18 years and they covered 14 prisons between them. Participants came from a variety of professions including nursing, dentistry, pharmacy, and psychiatry, and covered mental health, substance misuse and recovery, and user involvement. Several participants had managerial responsibilities.

The decision-maker group included nine females and five males aged between 37 and 59 years. One participant identified as being Black African and one as being African Caribbean. No decision-makers described themselves as having a disability or long-term condition. This group comprised a range of professions, including commissioners, governors and directors, and those with medical oversight. Together, the decision-makers we interviewed had responsibility for a huge portfolio of prisons with some individuals being responsible for up to 28 establishments. This meant there was good coverage of geographical regions, male and female populations, a range of security categories and specialist establishments (e.g. housing people convicted of sexual offences). We also spoke to a further two individuals who did not consent to a formal interview but provided valuable context. These meetings were not recorded and these individuals’ personal characteristics and words are not included in the findings section. We have withheld details of prison establishments and healthcare staff and decision-makers’ roles and job titles to protect participants’ anonymity.

We have organised our findings into three sections. Firstly, we explore the impact of the changes in healthcare on those living in prison, secondly we consider the impact on staff. Thirdly, we explore the theme of conflict, affording consideration to the tensions that arose within and between individuals and systems in response to the changes.

### The impact of changes to prison healthcare on people living in prison

Whilst each establishment had nuances to their delivery, most maintained urgent healthcare which included distribution of medication, blood clinics, emergency hospital visits and mental health support for those in crisis. However, most services were described as ‘drastically reduced’. Services operated by external agencies, such as podiatry or physiotherapy, were halted to reduce transmission of COVID-19 into the prison by visiting professionals.

Many services were delivered at cell doors to reduce prisoner movement around the prison and there was an increase in telephone and video triage and consultations. Remote care processes were not implemented consistently across prisons, however, and incompatibility between systems being used in prison versus the community reportedly complicated or prevented usage. Further, both clinicians and prison leavers felt strongly that remote consultations did not adequately replace face-to-face clinics. Participants said that services delivered at cell doors reduced healthcare professionals’ ability to adequately note changes in patient presentations, particularly when assessing mental health. They also expressed concerns over confidentiality and the limited time afforded to these appointments. As a result, prison leavers noted some were showing a reluctance to utilise these services, and some felt a dissatisfaction with the quality of care received when compared to face to face services.

Across the board, participants described dentistry as particularly difficult to access. Frontline staff and decision-makers explained that this was due to difficulties managing aerosol generating procedures. Most noted it was already challenging to access a dentist prior to the pandemic, but all prison leavers commented that it was now almost ‘impossible’.

*‘He actually got a razor blade and cut his own teeth out with his own gums because they told him he wasn’t going to get to see a dentist. He waited three days. He was crying… he actually cut his face open just to get the tooth out’ (Prison leaver).*

There was feedback from prison leavers, that non-COVID related healthcare was superseded by the implementation of a regime that prioritised security and infection management. At the height of the pandemic, this materialised as 23.5 hours behind cell doors, with prisoners often having to choose between a shower, exercise or a call to loved ones in the half hour out of their cell. This was accompanied by a reluctance amongst prison leavers to seek help. Reasons for this included a fear of overburdening healthcare staff (who were focused on COVID) and wanting to avoid a repeat negative experience where healthcare concerns had been minimised. Several mentioned that infection control measures had meant an increase in the provision of paracetamol in place of a full medical appointment.

*‘You’re just getting ignored because the ultimate thing was literally, “Why are you here? Is it urgent? Have a paracetamol.” A paracetamol doesn’t solve everything’ (Prison leaver).*

Healthcare staff remarked they could not undertake ‘social prescribing’ either, encouraging people to take part in exercise or meaningful activities as an alternative to medication.

Those with mental health needs were reportedly disproportionately affected. In every prison leaver interview, participants spoke of the psychological impact of the lockdown to prisoners, and emerging mental health needs for either themselves or others. The experience was described as traumatic and deeply impactful. Prison leavers and staff described how the reduced regime and face-to-face contact not only resulted in emerging or worsening needs but also meant there was less opportunity to visually observe the psychological impact of the pandemic on prisoners, to note withdrawal or distress, or even self-injury.

*‘We also had six people take their own lives across [our] prisons through suicide during the COVID pandemic and I think we lost four people to COVID. So not only, you know, like the community, not only were we dealing with everything that was going on, we were losing people because of their mental health issues and they couldn’t cope’ (Frontline healthcare staff).*

Prison leavers remarked on how the decline in face-to-face contact also reduced their ability to seek ‘ad-hoc’ advice and receive informal care from healthcare staff on the wings. Several prison leavers described feelings of abandonment. Time behind their cell doors also reduced opportunities for informal peer support on health-related issues. One substance misuse worker reflected on the consequences of this in terms of self-medicating.

*‘A lot of the men didn’t know how to cope with that lack of routine so boredom crept in. I know last summer we saw quite a big spike in use of alcohol and cannabis, cocaine, [ecstasy]. … I think there’s been quite a lot of [Mandatory Drug Test] failures of prescription medication’ (Frontline healthcare staff).*

Specific groups of prisoners were reportedly more affected by changes to healthcare and the regime more than others. For example, psychiatric staff highlighted those with ADHD or personality disorder diagnoses as being significantly affected by the lack of social interaction and prolonged time in cell. In addition, those who could not read or write particularly well, and whose first language was not English were said to be underserved as they were unable to access support to communicate with healthcare teams.

Those who had non-urgent healthcare needs were deprioritised at the start of the pandemic, but were still waiting for an appointment at the point of interview, still listed as ‘not urgent’ in comparison to others. In a similar way, prison leavers reported the perception that young people were also disproportionately affected by the changes in healthcare as priority was given to seeing vulnerable (usually elderly) prisoners. Those who were already known to healthcare services before the pandemic, generally continued to have some healthcare service, while those who were below certain thresholds, found it harder to be seen than usual.

*‘I think obviously the prison were busy caring for the vulnerable and so those that wouldn’t be classed as vulnerable suffered...*, s*o those that were vulnerable were getting that bit more care and those that were less vulnerable were getting even less care than they were getting before’ (Prison Leaver).*

Despite recognising significant effort from individual healthcare professionals, prison leavers conveyed the sense that prison healthcare services could not meet demand due to the constraints caused by the pandemic,staff shortages, and inadequate space.

Some prison leavers reported receiving treatment in the community upon their release for issues they had been denied help for in prison. This, with delayed intervention, was reported to have consequences for their health. For example, one participant on release was diagnosed with diabetes that she had been reporting symptoms of, but had not been seen for, whilst in prison. Another stated their community GP was unable to access any records from the prison relating to their newly diagnosed condition, as the prison was not responding to requests.

Aside from unmet physical health needs, reduced healthcare staff and face-to-face appointments also affected prisoners’ access to ad-hoc care. Prison leavers reported the loss of valued aspects of care such as informal conversations, group-work and wellbeing checks, which contributed to their overall sense of health and wellbeing. Moving forwards, artefacts remain due to changes to prison healthcare during the pandemic.

*‘The waiting lists are now enormous and also having to go to hospital for scans and things. Like all of those things have now got huge wait lists which probably are in-line with some of the stuff in the community but I think prisoners don’t necessarily understand it.’ (Frontline healthcare staff).*

These lengthy waiting lists will affect healthcare in the future. So too will increased mental health problems in prisoners, and the weight gain and musculoskeletal needs caused by immobility. These will contribute to the long-term impact of COVID-19 restrictions on prisoners. Finally, it is useful to note that instances of good practice and innovation were raised within our interviews. One example is of the erection of ‘pods’ in the grounds of the establishment for shielding prisoners. Another was the temporary implementation of 24-hour healthcare, which was done to reduce the demand on officers in terms of night escorts. One further example in one establishment was a weekly TV broadcast to keep prisoners informed of the current situation and conditions in the prison.

### The impact of changes to prison healthcare on people working in prison

The early weeks and months of COVID-19 were described by decision-makers and frontline healthcare staff as periods of mass uncertainty. Prisoners were looking to healthcare and operational staff for information, healthcare staff were looking to their commissioners and governors, who in turn were looking to NHS England, Public Health England, HMPPS and the British Government. Information was described as changeable and, at times, frightening. One decision-maker talked about the horror of ordering body bags at the start of the pandemic, not knowing how many deaths there might be from COVID-19. The healthcare staff we spoke to felt vulnerable and even traumatised by the experience. The clearest message from interviews with frontline staff and decision-makers was that the workforce were completely exhausted by the pandemic given their efforts and commitment to maintain a minimum level of provision throughout.

*‘We are knackered. We are absolutely on our knees’ (Frontline healthcare staff).*

*‘I had literally had no days off for 16 months’ (Decision-maker).*

Frontline healthcare staff consistently relayed their unwavering sense of duty to the patients in their care but also reflected on the challenges associated with providing an adequate service, whilst observing restrictions and mandates from local and national management. Pre-March 2020, prison healthcare was already understaffed. The pandemic brought further complexity and uncertainty around shielding, self-isolation following contact with a suspected or confirmed COVID case, and (where applicable) homeworking. This affected both healthcare staff and prison officers, with implications for healthcare.

‘*So when you’re losing your workforce everybody is becoming tired and, you know, because do you remember in the beginning it was 14 days you had to isolate then that changed to 10 days but we had like 11 nurses off in one day in one prison, that’s quite a lot for a prison. That might be okay in a hospital but it’s not in a prison and of course you couldn’t cross people from prison to prison’. (Decision-maker).*

Prison officers are responsible for passing on paper and verbal applications by prisoners for appointments, and for unlocking cell doors and escorting prisoners to appointments. Consequently, prisoners’ ability to communicate with healthcare staff, request appointments, and access clinics and appointments was affected.

Frontline staff also described fatigue due to undertaking their usual work with fewer staff plus additional Covid-related tasks, such as vaccinations, testing, attending COVID boards, compiling and updating risk registers and providing weekly COVID figures. Some described the level of expectation as unreasonable.

*‘All of a sudden you have an outbreak and then you’re doing mass testing. So you’re mass testing a whole prison or a whole wing, so how are you supposed to do something else as well?’ (Frontline healthcare staff).*

Others described confusion over whose responsibility certain jobs were, with healthcare often taking on the task as the default position.

*‘I think it was the fact that for quite a long time nobody actually had responsibility for testing. There was no kind of agreed process between PHE, HMPPS, Department of Health and Social Care and nobody knew where it sat. So the pressure just kept getting put on healthcare and we didn’t have the resources to do it but, you know, even if you put funding in place we don’t have the staff available to do it.’ (Decision-maker).*

Staff expressed frustration about all covid-related tasks being allocated to an already stretched healthcare team such as distributing food to COVID patients’ cells. The combination of frustration and tiredness together resulted in a degree of bitterness for some. Those working at home described feeling ‘guilty’ or ‘bad’ about not being on the frontline yet were also fatigued. However, they were reluctant to complain as they were making comparisons with their prison-based colleagues.

*‘I’ve had an outbreak meeting for every prison, five days a week. I was working from seven am until eight every night, every day, plus I had my healthcare meetings individually, you know, we had infection, prevention control meetings. We had quality meetings. Still had to do my contract meetings. It was just ridiculous.’ (Decision-maker).*

COVID infections in the prisons appeared to peak in most establishments in Autumn of 2020, when staff teams were already tired from the previous 6 months and needing time to pause and re-energise. The sheer numbers of COVID at that time meant this was impossible.

*‘B wing was the elderly and vulnerable wing and had 500 people on it. So we tested everybody and within three days we got all the results back and I think we had something like 440 people all positive on one wing’ (Decision-maker).*

Some prison leavers mentioned they had noticed stress and tension in staff towards the latter half of 2020.

*‘There is no question that that had a massive impact on them, do you know what I mean and you could tell like with half their attitudes, you know, their heads went down, you know, you could tell that they were just tired. I mean it hit them hard because, you know, obviously their numbers dwindled, so the nurses they were off and whatever, so you had a few of them doing a job of many’ (Prison leaver).*

The impact of the pandemic on prison healthcare staff represents a clear and continued risk to prison healthcare, as summarised by one decision-maker:

*‘Each individual institution relies on its workforce and without workforce there isn’t a health care service and if you can’t look after the health workforce. I understood suddenly how important the health workforce is; their sickness, how they feel about things, listening to them, understanding what’s happening for them, giving them the correct PPE. I understood that you need to protect them. Look they’ve got it hard, support is what they need in my commissioner role. This isn’t the time to try and poke them with a stick and say, come on you could be doing more. If they’re managing to get into work under these circumstances, they’re doing a good job’ (Decision-maker).*

### Discord, divergence and distress

We heard narratives of trauma across all groups we interviewed, with the pandemic having a palpable impact on everyone but in divergent ways at distinct stages of the pandemic. Participants’ experiences appeared to converge during the early months of the pandemic, with a sense of ‘collective spirit’ and that ‘everyone was in it together’. However, this unravelled into ‘collective despair’ creating chasms within teams and between prison leavers and staff. Discord was a recurrent notion across and between all participant groups. Two main and important areas of conflict arose. The first was between prisoner and staff experiences and the second was between how prison healthcare staff perceived that they were viewed and treated compared to their counterparts in the community. Prison leavers and staff conveyed their impression that as the pandemic progressed, the compounding effect of COVID-19 on prison healthcare appeared to create a ‘race to the bottom’ and an unavoidable sense of competition between teams based at different prisons and between community and prison healthcare.

#### Prisoner and staff experiences: neglected versus exhausted

The conflict between prisoner and staff experiences arose out of prisoners’ feeling their health needs were neglected whilst staff reported feeling exhausted but doing their best. Participants’ accounts were littered with juxtaposition. Prison leavers, for example, described a sense of feeling abandoned, frustrated by the lack of face-to-face healthcare and the length of time spent behind their cell doors. The number of staff physically present on site was reduced (to essential services only), while demand increased beyond usual expectations due to health needs related to lockdown and COVID infections. This created an unsatisfactory situation for all; from decision-makers to prisoners.

*‘I feel like when the pandemic came….they were barely there and I think at that stage a lot of people were becoming ill within the establishment. So whether it be mental illness or physical illness, there just wasn’t enough nursing staff there or health care staff and when they was there because there was so many people wanting to see them, there just wasn’t enough time in one day to see the people and they’d probably come in once or twice in the week because of the pandemic and when they did come in, they would probably be like one doctor and two nurses and there’s like 400 to 600 prisoners in one prison. So yeah the maths just doesn’t make sense’ (Prison leaver).*

There was an interesting juxtaposition between the perception of the vast majority of prison leavers, who overwhelmingly had a sense that ‘everything stopped’, and frontline practitioners and decision-makers, who spoke about the flurry of activity at the start of the pandemic.

*‘How do we make sure that people are getting their medication? What if somebody needs to be seen? How do we just keep a track on people who ordinarily you’d be doing these five things. You can’t. What about keeping people safe in their cells if they’re locked up for really long periods of time?’ (Decision-maker).*

The activity taking place within healthcare teams was necessary if they were to manage the needs of the prison, but also because of changing guidance from the top. However while this was happening, prisoners reported being largely uninformed, which contributed to stress at the time.

Several prison leavers expressed gratitude towards healthcare staff, highlighted good practice and recognised that healthcare staff had put themselves at risk.

*‘... they tried, the healthcare they tried, you know, at least for being able to arrange for you to be able to speak to a doctor over the phone even if you’re not seeing them physically and the nurses, medications were still given out. So even if there is a bit of a delay, they still tried. I heard of some instances where there was some COVID in the prison but the nurses were still there, you know, they were seeing people. So they deserve the praise’ (Prison leaver).*

*‘The nurses were really overworked and overwhelmed and that. They were putting themselves in danger every day. I think that health care was probably the only people that seemed to really care what was going on with people in custody at the pandemic, like the staff became really lazy that worked on the wings and I think health care were the heroes in my eyes’ (Prison leaver).*

The overarching narrative throughout interviews was one of *‘you tried your best but we still suffered enormously*’ and many staff acknowledged this.

*‘The men in this prison have been outstanding. I cannot tell you how tolerant they have been. How compassionate to staff they have been and how brilliantly they’ve worked alongside us. I don’t doubt for a second that the impact on them is much greater than we could possibly know and I think that’s yet to still be seen’. (Frontline healthcare staff).*

#### Prison versus community healthcare

Staff also expressed feeling ‘neglected’ and ‘left’. Many worked in settings that were not conducive to running efficient healthcare services, such as nineteenth century healthcare buildings which prevented services getting back up and running post covid (e.g. dental suites). Others mentioned the frustrations and challenges of requiring escorting officers to move around the secure environment, particularly given the number of operational staff on sick leave or self-isolation.

*‘Even if we had all the health care staff available it didn’t necessarily mean that we had all the prison staff available to unlock and supervise’ (Frontline healthcare staff).*

Additionally, prison healthcare staff suggested that the rules and directives about keeping people apart and face-to-face contact to a minimum upset the balance required to provide prisoners with appropriate healthcare, resulting in unmet need.

*‘I sort of spoke up and said, actually I think, as much as I understand what we’re trying to achieve and I fully support that because I want to keep everybody safe. I think there still needs to be a presence of some sort because if we have someone in crisis, we as professionals in that area, need to be here.’ (Frontline healthcare staff).*

Healthcare staff also described tension arising out of the level of demand placed on them compared with community teams who were able to bring in additional resources for the pandemic-related tasks. One healthcare provider described feeling ‘abandoned’.

*‘In the prisons our healthcare teams are vaccinating and they are testing. In hospitals their staff aren’t also having to go and vaccinate somebody. They aren’t also having to test’ (Decision-maker).*

Whilst commissioners and decision-makers described a sense of improved joint-working and communication ‘at the top’, frontline healthcare staff felt they did not benefit from this leading to cracks in the initial collaborative effort of external teams.

*‘I think there was some conversation about being able to access community support for that but I didn’t see any prison actually manage that even when they approached the community. There wasn’t that support of anybody coming in to support them’ (Decision-maker).*

Prison leavers also drew comparisons between the quality of prison and community healthcare.

Participants from all groups conveyed the sense that prisons were a low priority and not as important as other communities.

*‘We were a week behind other prisons and who had less vulnerable people and yet we were not prioritised. I did feel that we were probably, our prisoners and their sentences [for sexual convictions] were probably in people’s minds when they were prioritising who should get a vaccination. I mean I might be wrong but that’s how it felt’ (Decision-maker).*

Participants particularly noticed the divergence between prisons and the community as the community started to ‘get back to some sort of normality’ in the summer of 2020 while prisons did not. Prison leavers, decision-makers and front-line staff all said they felt left behind. For example, staff mentioned inadequate resourcing, including basic infection control equipment such as personal protective equipment (PPE) in the early months, and prison leavers commented on the lack of sanitisation despite the posters and government broadcasts about this being of paramount importance. They noted the contradiction between the stark restrictions to the regime for the purpose of infection control, and the absence of requirements for operational staff to wear PPE.

## Discussion

In this study, we found evidence to suggest that prison healthcare has been transformed by the COVID-19 pandemic with potential long-term implications. We identified key changes including additional responsibilities on staff, the unavailability of appointments (especially face to face), modified methods of delivery, growing waiting lists and the associated deterioration in the health of those coordinating, delivering and receiving healthcare services. Participants from all three groups described being significantly impacted by their experience of the COVID-19 pandemic, although in vastly different ways.

Healthcare staff, both on the front-line and commissioning services were affected by the level of work required to maintain a minimum service. Much of this theme is connected to the additional barriers that prison-based healthcare teams had to overcome as a result of the legacy factors, if patients’ needs were to be adequately addressed. Importantly, workforce stress has the potential to impact on staff attitudes, delivery of care and retention of staff, all of which are crucial for the continued provision of healthcare. Many participants were demonstrating signs of burnout or explicitly referring to it. ‘Burnout’ is a syndrome resulting from chronic workplace stress, showing itself through exhaustion, distancing oneself from one’s job, and feelings of negativity or cynicism (De Hert, 2020). Our findings mirror concerns about the wellbeing of NHS staff in the community. A 2020 survey with almost 600,000 NHS workers found 44% to be suffering with work-related stress (up to 50% for those who had worked on a COVID-19 specific ward or area during 2020). Around one third of those who responded said they had considered quitting their job (O’Dowd, 2021). Our findings showed that prison healthcare teams (and decision-makers to some extent) found themselves at a disadvantage to community teams, which was a source of both disappointment and frustration. They described having fewer resources (including personnel and space), increased responsibilities, and less recognition from the public. They were also working at a time where high numbers of operational staff were off-sick (Hewson et al., 2021), generating even further complications to seeing prisoners.

Changes to healthcare provision were just one of many changes experienced by prisoners during the pandemic. Although our interviews focused on healthcare, prison leavers highlighted the holistic nature of health and indicated that emotional *and* physical wellbeing was impacted by changes to the regime. For example, prisoners endured prolonged isolation (with most still locked up 22 hours a day at the end of 2021), prevention of physical visits for at least a year and the termination of purposeful or rehabilitative activity (Prison Reform Trust, 2022). Consequently, prisoners reported weight gain and muscle wastage from a lack of movement and the provision of additional food described as high in fat, calories and salt (Gipson & Wainwright, 2020; Prison Reform Trust, 2021; Suhomlinova et al., 2021). The United Nations defines solitary confinement as “the confinement of prisoners for 22 hours or more a day without meaningful human contact” and describes prolonged solitary confinement (beyond 15 days) as “cruel, inhuman, or degrading” (United Nations Office on Drugs and Crime, 2015). Solitary confinement can cause anger, depression, anxiety, paranoia, psychosis, and aggravate pre-existing mental illness (Shalev & Edgar, 2015). Through the pandemic, prisoners have endured almost total confinement, often solitary.

Prisoners did not see family or friends which can help alleviate stress, and the lack of communication from some prisons and healthcare teams which we heard about from some participants has been highlighted as a ‘soft power’ adding further emotional strain to prisoners (Maycock, 2021). Suhomlinova et al. (2021) analysed prisoners’ lived experiences of the pandemic through correspondence, and detail a range of harms to prisoners’ mental health including increased incessant noise from prisoners banging their walls, lethargy, self-harm, irritability and a withdrawal from the limited social opportunities on offer. Maycock (2021) argues that COVID-19 has eroded hope in prisoners. Certainly, given what was known about the mental health needs of people in prison *before* the pandemic, and the enduring effects of prisons on wellbeing (Durcan, 2021; Prison Reform Trust, 2022), and the emerging findings around mental health of prisoners since (Johnson et al., 2021), this is of significant concern. While many people in the community may also have felt frustrated at a lack of face-to-face healthcare provision, prisoners simply had no alternative options when the answer was ‘no’ to being seen or accessing treatment. The lack of autonomy and freedom is an expected and integral part of imprisonment, but in ordinary times the psychological effects are mitigated through means such as physical exercise, family visits or personal development through courses or education. These have been severely limited (or prevented) for prisoners in the pandemic.

One estimate suggests that one fifth of adults across the general population of England will require long-term mental health support in the wake of COVID-19, for conditions such as PTSD, depression and anxiety (O’Shea, 2020). This has been echoed in recent work published by User Voice (2022). The intensity of the experience heard from those living and working in prison healthcare suggests there might be an even greater fall out for them than this already high estimate.

Prison Reform Trust (2021) referred to a continuum of experiences in relation to seeing a GP throughout the pandemic, starting with no GP appointments available, through limited provision, to ‘amazing’. Although we also found a broad range of experiences, we predominantly encountered reports of hidden health needs and challenges in access. The combination of existing (pre-Covid) security protocols with infection control measures meant other needs have been deprioritised. Patients already known to healthcare were prioritised while those whose healthcare concerns arose *during* the pandemic and/or were considered to be in a low risk group (e.g. young people) felt disadvantaged. This could indicate emerging health inequality and as such, aligns with Gipson and Wainwright (2020) findings.

Our data shows a sense of ‘collective spirit’ at the start of the pandemic, in line with evidence that people in prison had initially recognised restrictions to be ‘*necessary and proportionate’* (HM Chief Inspector of Prisons, 2021; Prison Reform Trust, 2021). However, we found that as the pandemic progressed, this sense of collective spirit culminated in collective hurt; a universal trauma, affecting everyone albeit in different, almost competing, ways. Some have questioned whether prisons should have prioritised the management of COVID-19 given the length of the pandemic and the pre-existing and worsening needs of prisoners (HM Chief Inspector of Prisons, 2021). Suhomlinova et al. (2021) conclude the balance was not appropriate and that staff-prisoner relationships have suffered from built-up frustrations and limited release outlets. Maycock (2021) also notes how the effort put into the early lockdown waned, giving rise to unresolved tension.

Despite living in the same storm, prison leavers, frontline staff and decision-makers were weathering this in very different boats. As the storm raged on, the interviews revealed a sense of comparison rather than community. The discussion section affords consideration as to how prison healthcare can return stronger, with such a fractured foundation.

### Strengths and limitations

It is a limitation of this study that data were collected from a self-selecting sample which may have impacted the narrative and subsequently, our findings. This was balanced, however, by the inclusion of participants with three distinct perspectives and, notwithstanding the conflicts highlighted in our findings, the commonalities we identified across these accounts. Furthermore, our findings support other work that, using different approaches, has charted changes to prison healthcare during the pandemic (Canvin & Sheard, 2021; Wainwright & Gipson, 2020; HM Chief Inspector of Prisons, 2021; Johnson et al., 2021; Prison Reform Trust, 2021). Also, although we have presented aggregate findings here, we did hear a diverse range of experiences which included positive and negative feedback.

The lived experience steering group played a key role in our development of themes, and our approach to interpreting and writing-up our findings. Specifically, discussions with the group led to the refinement of the theme of discord. The group were particularly interested in the fact that all parties reported being traumatised by the experience. They argued that given that prisoners’ experiences are usually denied and suppressed, that our reporting should not perpetuate this imbalance of power. They advised us to reconsider the comparability of prisoner and staff experiences and to ensure that the harm and distress experienced by prisoners was given due attention and not presented as equivalent to pressures faced by staff.

The topic was a particularly sensitive one and we encountered caution and resistance from some healthcare professionals and decision-makers, including two who declined to consent to a formal interview. Individuals who expressed caution explained that they did not want to be seen to be ‘whistleblowing’ if they reported challenges in prison healthcare delivery or how COVID-19 had been managed internally in the prison service. In contrast, those who agreed to participate often described the study as a cathartic opportunity to process and reflect on their experiences; an opportunity to be heard. Further, at the time of data collection (July to December 2021), COVID-19 was ongoing, with some prisons returning to reduced regimes, so it was a particularly busy period for study recruitment. We took account of this by allowing a long period for fieldwork to take place and offering weekend and evening appointments for interviews.

#### Implications and future research

This study was important in triangulating prisoners’ experiences of health and healthcare during the pandemic with professionals working through the pandemic. Our findings have important implications for how prison healthcare moves forwards, especially as a recent report suggests the prison system now faces a new storm of ‘rising prison numbers and a looming staffing crisis’ (Prison Reform Trust, 2022). Pre-March 2020, the prison healthcare system was imperfect and, in some places, already frail. Our data found that this has now been compounded by increased staff vacancies, a widening of prisoner health inequalities and increases in mental and physical health problems from prolonged confinement and reduced access to healthcare amidst a global pandemic. We know in the community, we are changed by COVID-19, and the prison population (staff *and* prisoners) is no different. It is imperative to recognise the implications and fallout of this period for all involved, from commissioners to managers, to front-line staff and prison leavers. The prevalent theme of conflict, indicates a need to reduce divergence and enhance convergence in an already fractured system. As we begin ‘living with COVID-19’, we also have to live with the *impact* of COVID-19. Within prison healthcare, this includes addressing health needs, health inequalities and the tensions experienced by all involved.

Future research could capitalise on hearing from front-line prison staff (such as Officers) to triangulate our findings with their experiences. Our research raised the following questions for decision-makers and future researchers:

- *How do we ensure that those who were disproportionately affected by changes to healthcare provision in prisons don’t continue to be so?*
- *How can you ‘build back better’, ensuring healthcare services are better than they were pre-pandemic, if you have a significantly fractured foundation?*
- *What can be put in place by way of managing mental and physical health in the absence or delays of seeing healthcare professionals such as peer support, groups and structured activities?*
- *How can healthcare services attract staff to fill vacant posts whilst supporting those who remain in post following the toll of COVID-19?*
- *How can the future demand on healthcare be best managed and supported, considering the backlog created by the pandemic? How can healthcare manage the repercussions of lockdown on people’s health and wellbeing and the resultant increases in demand? This is particularly pertinent for dentistry.*

Our findings suggest that many of the answers to these questions should be grounded in building relationships; amongst staff and colleagues, between prisoners and staff, repairing and reinitiating face-to-face contact and communication with them. It will be beneficial to understanding each others’ trauma and experience to bring back the collective spirit as we move into recovery. The focus now is less on survival, but on clearing up the debris that COVID-19 leaves in its wake. A multitude of prison leaver interviews indicated that the lack of human contact was one of the biggest impacts of COVID-19 on healthcare delivery. Therefore, it is important that, where possible, this is reinstated as a priority. To this end, relationships and connectedness can be seen to be key to longevity and long-term success of prison healthcare, not just for prisoner health but also staff retention and reductions in burnout. The research has also shown the benefit and importance of consulting multiple groups and ascertaining multiple perspectives; this approach should continue as we move towards recovery, affording consideration to the views of healthcare professionals and, imperatively, giving a platform and voice to prisoners, as to how healthcare should look moving forwards.

## Conclusion

This in-depth qualitative study has highlighted the impact of the changes that COVID-19 had on prison healthcare. It has afforded consideration to the experiences of three key groups working and living in prisons throughout the pandemic. The impact on prisoners and staff has been profound with long lasting implications. A key finding is the divergence this has caused between these two groups, despite an initial collective spirit. It is hoped that by shedding light on the perspectives of those involved in providing and receiving prison healthcare, communication and collaboration will be promoted as healthcare is restored.

## Data Availability

Unpublished data is not available.

## Footnotes

1 In command mode, individual prisons were described as a ‘bronze level’, nationally prisons were grouped together to report at a ‘silver’ level and a national COVID Gold command was formed to direct from the top. Exceptional Delivery Models referred to changes to regime, in response to current COVID outbreak status with a focus on prioritising and balancing meals, medication, prisoner safety and welfare and family contact with disease control.

2 The prisons included in this study were both private and statutory
